# Associations between breakfast eating habits and health-promoting lifestyle, suboptimal health status in Southern China: a population based, cross sectional study

**DOI:** 10.1186/s12967-014-0348-1

**Published:** 2014-12-11

**Authors:** Jieyu Chen, Jingru Cheng, Yanyan Liu, Yang Tang, Xiaomin Sun, Tian Wang, Ya Xiao, Fei Li, Lei Xiang, Pingping Jiang, Shengwei Wu, Liuguo Wu, Ren Luo, Xiaoshan Zhao

**Affiliations:** Department of Traditional Chinese Medicine, Nanfang Hospital, Southern Medical University, Guangzhou, Guangdong 510515 China; School of Traditional Chinese Medicine, Southern Medical University, Guangzhou, Guangdong 510515 China; Department of Rheumatic diseases, The First Affiliated Hospital, Guangzhou University of Chinese Medicine, Guangzhou, 510405 China

**Keywords:** Breakfast eating habits, Suboptimal health status(SHS), Healthy lifestyle, Cross sectional study

## Abstract

**Background:**

Suboptimal health status (SHS) is the intermediate health state between health and disease, refers to medically undiagnosed or functional somatic syndromes, and has been a major global public health challenge. However, both the etiology and mechanisms associated with SHS are still unclear. Breakfast eating behavior is a dietary pattern marker and previous studies have presented evidence of associations between failure to consume breakfast and increased diseases. Accordingly, in view of the significance of breakfast eating behaviors with respect to health status, the associations between breakfast eating habits and healthy lifestyle, SHS require further elucidation.

**Methods:**

A cross-sectional survey was conducted within a clustered sample of 24,159 individuals aged 12–80 years in 2012–13 within the population of Southern China. Breakfast eating habits were categorically defined by consumption frequency (‘*scarcely, sometimes or always*’). Health-promoting lifestyle was assessed via the *health-promoting lifestyle profile (HPLP-II)*. SHS was evaluated using the medical examination report and *Sub-health Measurement Scale V1.0 (SHMS V1.0).*

**Results:**

Of the 24,159 participants, the prevalence rates for the ‘health’ , ‘SHS’ , and ‘disease’ were 18.8%, 46.0%, and 35.2%, respectively. Overall, 19.6% of participants reported ‘scarce’ breakfast eating habits, with frequent breakfast eaters scoring higher on both *HPLP-II* and *SHMS V1.0*. After demographic adjustment, regression analyses revealed a significant association between breakfast eating habits and healthy lifestyle (p <0.001). There were lower levels of breakfast consumption regularity amongst individuals with SHS than those with disease. Categorically ‘scarce’ breakfast eaters were approximately three times more likely to be assigned SHS (OR: 2.745, 95% CI: 2.468-3.053), while infrequent breakfast eaters (‘sometimes’) were just less than twice as likely to be assessed as being of SHS (OR: 1.731, 95% CI: 1.595-1.879).

**Conclusions:**

Breakfast eating habits are significantly associated with a healthy lifestyle, and appear to be a useful predictor of a healthy lifestyle. Irregular breakfast eating habits are related to an increased risk of SHS; increased breakfast eating frequency may contribute to lowering the prevalence of SHS in Southern China.

**Electronic supplementary material:**

The online version of this article (doi:10.1186/s12967-014-0348-1) contains supplementary material, which is available to authorized users.

## Background

It has been regularly and widely propounded that breakfast is the most important meal of the day; breakfast consumption has been shown to improve energy, control appetite and reduce the risk of overeating [1,2]. Breakfast eating behavior is considered a dietary pattern marker, in addition to an essential healthy lifestyle component [3,4]. Previous studies have presented evidence of associations between failure to consume breakfast and increased bodyweight [5,6], in addition to contraction of cardiovascular disease [7], metabolic conditions [8,9], dyslipidemia and insulin resistance [10], type 2 diabetes mellitus [11] and reproductive dysfunction [12]. Moreover, regular breakfast consumption has been correlated with energy balance [13], behavioral and cognitive functioning [14,15], personal wellbeing and mental health [3,16]. Furthermore, significant relationships between health status and lifestyle have been evidenced, as underscored with the importance of breakfast eating due to the concurrent positive effects on health status [17,18].

In parallel with social economic development and the increasing pace of life, a growing appreciation for the importance of health has developed. The overarching concept of health status has been categorized into three distinct types, namely health, disease and the intermediate state between health and disease, referred to as suboptimal health status (SHS). SHS refers to medically undiagnosed or functional somatic syndromes [19-22], characterized by a decline in vitality, physiological function and the capacity to adapt to varying conditions [23]. People in SHS frequently suffer from symptoms including chronic fatigue, headaches, dizziness, depression, anxiety, non-specific pain (e.g. back pain and chest pain), functional system disorders (e.g. digestive system, cardiovascular system, respiratory system, urinary system). Accordingly, SHS sufferers are typified by impaired quality of life, frequent hospital visits and incurred medical expenses [20,21]. A previous investigation by the current authors found that suboptimal health status was applicable to 65.1% of the surveyed population in Southern China [24], with SHS now a major global public health challenge [20,25,26]. Current prevention and intervention strategies recommend disease prevention and effective treatment of early-stage illness [27,28]. However, both the etiology and mechanisms associated with SHS require further elucidation.

Accordingly, in view of the significance of breakfast eating behaviors with respect to health status, the authors theorize that breakfast eating habits are likely related to SHS. To date, no human studies of breakfast eating behaviors with respect to SHS have been published, in concurrence with a paucity of data pertaining to the potential association between breakfast eating habits and health status within the Chinese population. Thus, a comprehensive cross-sectional study was conducted within Southern China which aimed to examine the existence of associations between breakfast eating habits, healthy lifestyles and the risk of SHS.

## Methods

### Study design and population

The study was a cross-sectional descriptive survey, with a three-stage stratified sampling method employed. In stage one, six areas of South China (Guangzhou, Zhuhai, Huizhou, Jiangmen, Zhanjiang, and Shaoguan) were selected to be representative of economic characteristics, population demographic and geographic distribution. In the second stage of sampling, one region was randomly selected from each of the aforementioned areas. In the final stage, one unit (e.g. schools, companies, government agencies or factories) was selected from each region.

Overall, 28,144 individuals aged 12–80 years from 14 primary sampling units undertook the baseline survey in 2012–13, with 24,159 individuals (11,796 men and 12,363 women) included for the current analysis. Due to the invalid responses information (e.g. breakfast eating habits, baseline characteristics or the scale written in chaos), 3,985 respondents were excluded from further consideration, resulting in a valid response rate of 85.8%. Informed oral consent was obtained from every participant prior to data collection. Verbal consent was deemed sufficient as participants had previously volunteered for the study and could refuse to participate. All data were kept strictly confidential. The ethics committee also approved the consent procedure.

### Survey instrument

The developed questionnaire was a combination of self-designed questionnaire items and a standardized questionnaire. The self-designed questionnaire components comprised the general demographic characteristics (including age, gender, BMI, married status, education level, occupation, smoking, and drinking) and breakfast eating frequency assessment, defined according to frequency of behavior using a three-point respond format, “scarcely, sometimes, always”, with the rating score ranging from 1 to 3. The standardized components were based upon the “*Sub-Health Measurement Scale V1.0 (SHMS V1.0)*” and the “*Health-promoting lifestyle profile (HPLP-II)*” to permit assessment of participants’ health status and health-promoting lifestyles. Uniform instructions were provided by trained investigators, with questionnaires self-completed by participants over a 30 minute period.

### SHS assessment

Health status assessment was performed in accordance with a medical examination report and SHMS V1.0, according to clinical guidelines for SHS published by the China Association of Chinese Medicine [23,29]. SHMS V1.0 was developed by our research group, with Chinese research data indicating that the scale has a high level of reliability and validity, with a Cronbach α and split-half reliability coefficients of 0.917 and 0.831, respectively [30]. The scale consists of 39 items in total, 35 of which are divided among three symptom dimensions (physiological symptoms - 14 items, psychological symptoms - 12 items and social symptoms - 9 items). Participants were asked about uncomfortable symptoms that they had experienced during the previous month, with total scores then calculated. A low total score represents a high likelihood of SHS (i.e. poor health). Prior to surveying, participants had attended an annual unit health examination in hospital, comprising medical history, a physical examination, blood haematology and biochemical analyses, rest ECG and chest radiography. After exclusion of participants diagnosed with clinical disease in the health examination by clinical doctors, threshold values for SHS within the physiological, psychological and society dimensions of SHMS V1.0 were 68, 67 and 67, respectively. If participants were not in SHS with respect to any of these three dimensions (physiological, psychological and society), they were considered healthy [23,30].

### Lifestyle health assessment

HPLP-II has been widely used as a measuring tool for assessing health promoting behaviors and is considered to offer both reliability and validity, domestically and internationally [31,32]. It comprises 52 items assessing six dimensions of lifestyle: self-realization, health responsibility, sports and exercise, nutrition, interpersonal relationship and stress management. It may be used to assess the frequency of the health promoting behaviors, based on a self-reported four-point Likert scale: “never, sometimes, usually, always”, with the rating score ranging from 1 to 4. The minimum and maximum HPLP-II scores are 52 and 208, respectively. The higher score represents a maximal level of health with respect to lifestyle. Health promoting lifestyle score is divided into four grades: poor (52–90), general (91–129), good (130–168) and excellent (169–208).

### Statistical methods

Descriptive statistics have been presented as frequencies. Univariate analyses were used to compare varying breakfast eating frequencies with both lifestyle health and health status scores using one-way ANOVA and Bonferroni correction for ad-hoc multiple comparisons. Multinomial logistic regression was used to estimate the typical profile for irregular breakfast eaters based upon demographic variables. The reference group was defined as those participants with the lowest level of exposure i.e. habitual breakfast eaters, or excellent lifestyle behavior. Multinomial logistic regression was used to estimate associations when using lifestyle health and health status as the outcomes of interest. Adjustments were made for potential confounders within multivariable models based upon collated demographic data. Estimation of the odds associated with self-reported symptoms of SHMS V1.0 was undertaken by adding breakfast eating frequency and demographic adjustments to multinomial logistic regression. The percentage of participants with missing values associated with relevant covariates was small e.g. 1.0% for smoking, and 0.7% for alcohol consumption; accordingly, all participants with available data for all covariates were included for multivariable analysis. All data analyses were done using SPSS 13.0. All p-values were two sided, with values <0.05 considered statistically significant.

## Results

### Sample characteristics

Baseline characteristics of all study participants are presented in Table 1. Of the 24,159 participants (11,796 men and 12,363 women), the mean age was 27.07 years. The prevalence rates for the ‘health’ , ‘SHS’ , and ‘disease’ groups of participants were 18.8% (4,533), 46.0% (11,121), and 35.2% (8,505), respectively. The major diseases that were reported affected the respiratory, digestive systems and endocrine or autoimmune systems, such as chronic rhinitis (10.6%), chronic pharyngolaryngitis (9.4%), chronic gastritis (4.7%), gynaecopathia (4.3%), chronic insomnia (3.8%), haemorrhoids (3.5%), breast disease (3.1%), fatty liver (2.3%), hypertension (2,0%), chronic bronchitis (1.8%), gastroduodenal ulcer (1.8%), rheumatic disease (1.8%), hyperlipemia (1.3%), chronic joint disease (1.2%), chronic otitis media (0.9%), thyroid disease (0.8%), cholecystitis (0.6%), chronic hepatitis (0.5%), heart disease (0.5%), diabetes (0.4%), prostatic diseases (0.4%), cancar (0.4%), asthma (0.3%), cerebrovascular disease (0.2%), arteriosclerosis (0.1%), tuberculosis (0.1%), chronic nephritis (0.1%).

**Table 1:** **Baseline characteristics of participants (n = 24,159)**

**Characteristic**	**n**	**%**
Age		
≤25	13,759	57.0
<25 and < =35	5114	21.2
>35	5286	21.9
Gender		
Man	11,796	48.8
Woman	12,363	51.2
BMI		
Non-overweight	21,735	90.0
Overweight	2424	10.0
Married status		
Unmarried	14,358	59.4
Married	9801	40.6
Education level		
Less than junior high school	2389	9.9
High school or college	8848	36.6
Bachelor degree or above	12,922	53.5
Occupation		
Professional	5370	22.2
Manager	3627	15.0
Clerk	233	1.0
Soldier	11	0.0
High school students	10,178	42.1
Production personnel	131	0.5
Operating personnel	2577	10.7
Business, service personnel	360	1.5
Freelancer	873	3.6
Inoccupation (housewife, etc.)	56	0.2
Others	743	3.1
Smoking		
No	20,409	84.5
Yes	3251	14.5
Quit	260	1.1
Missing	239	1.0
Drinking		
Never	7157	29.6
Little	10,925	45.2
Sometimes	5316	22.0
Often	555	2.3
Always	40	0.2
Missing	166	0.7

### Profile of irregular breakfast eaters

Baseline population demographics of the group characterized by irregular breakfast eating are presented in Table 2. Results indicate that demographic characteristics are significantly associated with irregular breakfast eating, particularly amongst those reporting the lowest level of breakfast eating frequency. Participants who reported poor breakfast eating habits were more likely to be young, male, unmarried, have a lower level of formal education and higher levels of smoking and drinking.

**Table 2:** **Odds ratios pertaining to irregular breakfast eating habits for baseline demographic characteristics via binary logistic regression modeling**

**Independent variables**	**Scarcely**			**Sometimes**		
	**B** ^**a**^	**OR (95% CI)**	**p-value**	**B** ^**a**^	**OR (95% CI)**	**p-value**
Age						
≤25	0.668	1.950 (1.633-2.329)	0.000	0.260	1.297 (1.104-1.523)	0.002
<25 and < =35	0.388	1.474 (1.311-1.657)	0.000	0.169	1.184 (1.074-1.306)	0.001
>35	Reference			Reference		
Gender						
Man	0.564	1.757 (1.618-1.908)	0.000	0.307	1.359 (1.269-1.456)	0.000
Woman	Reference			Reference		
Married status						
Unmarried	0.495	1.640 (1.425-1.888)	0.000	0.284	1.329 (1.167-1.513)	0.000
Married	Reference			Reference		
Education level						
Less than junior high school	0.431	1.538 (1.345-1.758)	0.000	0.461	1.585 (1.409-1.784)	0.000
High school or college	0.218	1.243 (1.138-1.358)	0.000	0.234	1.264 (1.175-1.361)	0.000
Bachelor degree or above	Reference			Reference		
Occupation						
Professional	0.549	1.732 (1.506-1.992)	0.000	0.018	1.018 (0.896-1.157)	0.781
Manager	0.690	1.994 (1.715-2.319)	0.000	0.100	1.105 (0.964-1.266)	0.153
Clerk	0.836	2.308 (1.622-3.283)	0.000	0.010	1.010 (0.725-1.407)	0.953
Soldier	−0.085	0.919 (0.165-5.106)	0.923	0.342	1.408 (0.375-5.292)	0.612
Production personnel	1.242	3.462 (2.143-5.594)	0.000	0.567	1.763 (1.117-2.784)	0.015
Operating personnel	0.737	2.090 (1.807-2.417)	0.000	0.355	1.426 (1.250-1.628)	0.000
Business, service personnel	1.463	4.320 (3.247-5.749)	0.000	0.574	1.775 (1.338-2.355)	0.000
Freelancer	1.050	2.858 (2.347-3.480)	0.000	0.391	1.479 (1.223-1.789)	0.000
Inoccupation (housewife, etc.)	1.335	3.799 (1.939-7.445)	0.000	0.321	1.378 (0.691-2.749)	0.363
High school students	Reference			Reference		
Drinking						
Yes	0.286	1.331 (1.220-1.452)	0.000	0.281	1.324 (1.235-1.420)	0.000
No	Reference			Reference		
Smoking						
Yes	0.498	1.646 (1.481-1.828)	0.000	0.155	1.167 (1.056-1.290)	0.002
No	Reference			Reference		

^a^Unstandardized regression coefficients.

The model is adjusted for demographic variables include age, gender, BMI, married status, education level, occupation, drinking and smoking.

### Breakfast eating frequency compared with health-promoting lifestyle and health status

Results of one-way ANOVA for breakfast eating frequency compared with both health-promoting lifestyle and health status are presented in Table 3. Those who reported the lowest level of breakfast eating frequency accounted for 19.6% of the total sample population. Analyses revealed significant differences with respect to six dimensions of health-promoting lifestyle and breakfast eating frequency, in addition to the total *HPLP-II* score (F _(2, 24156)_ = 1738.884, P = 0.000). After Bonferroni correction for multiple comparisons, this difference was statistically significant (p <0.001). Conversely, habitual breakfast eaters scored significantly higher on health status scales and the total *HPLP-II* score (F _(2, 24156) =_ 502.081, P = 0.000). Bonferroni ad-hoc tests revealed significant differences with respect to breakfast eating frequency (p <0.001).

**Table 3:** **Breakfast eating frequency compared with health-promoting lifestyle and health status using one-way ANOVA**

	**Breakfast eating frequency**			**One-way ANOVA**		
	**Group 1: scarcely (n = 4,738)**	**Group 2: sometimes (n = 7,285)**	**Group 3: always (n = 12,136)**	**F value**	**P-value**	**Multiple comparisons**
Health-promoting lifestyle						
Self-realization	22.36 (5.36)	24.16 (4.69)	26.02 (5.09)	969.296	0.000	G1 <G2 <G3^**^
Health responsibility	14.88 (5.49)	15.96 (5.79)	16.94 (6.35)	211.507	0.000	G1 <G2 <G3^**^
Sports and exercise	15.24 (4.27)	16.39 (4.33)	17.51 (5.07)	425.692	0.000	G1 <G2 <G3^**^
Nutrition	15.42 (3.35)	17.75 (3.27)	19.89 (3.86)	2791.141	0.000	G1 <G2 <G3^**^
Interpersonal relationship	21.30 (4.61)	23.19 (3.94)	25.12 (4.48)	1408.129	0.000	G1 <G2 <G3^**^
Stress management	18.28 (3.92)	20.13 (3.48)	21.90 (4.12)	1563.43	0.000	G1 <G2 <G3^**^
Total score	110.98 (19.90)	121.25 (18.68)	131.10 (21.84)	1738.884	0.000	G1 <G2 <G3^**^
Health status						
Health (n = 4,533)	79.60 (5.65)	79.60 (5.37)	81.15 (6.01)	38.627	0.000	G1 <G2 <G3^**^
SHS (n = 11,121)	62.60 (8.82)	64.29 (7.85)	66.08 (7.41)	171.653	0.000	G1 <G2 <G3^**^
Disease (n = 8,505)	59.89 (10.93)	63.06 (10.22)	65.60 (10.52)	175.604	0.000	G1 <G2 <G3^**^
Total score	63.78 (10.98)	66.46 (10.26)	69.30 (10.64)	502.081	0.000	G1 <G2 <G3^**^

Data presented as mean (SD). ANOVA indicates analysis of variance. Bonferroni was used in the multiple comparisons.

^**^ *P* <0.001 (Significant after Bonferroni correction for post-hoc analysis).

### Associations between breakfast eating frequency and health-promoting lifestyle

Associations found between breakfast eating frequency and health-promoting lifestyle are presented in Table 4. Multivariable regression analyses with adjusted demographic variables revealed a significant association between breakfast eating frequency and health-promoting lifestyle (p <0.001). Compared with habitual breakfast eaters, those with the lowest breakfast eating frequency were approximately 95 times more likely to exhibit a poor health-promoting lifestyle (OR 95.415, 95% CI 58.489-155.654), while those that infrequently ate breakfast were almost 12 times more likely to exhibit a poor health-promoting lifestyle (OR 11.855, 95% CI 8.839-15.900). Furthermore, skipping or infrequent breakfast eating had marked associations with respect to other components of a healthy lifestyle including poor nutrition (OR 193.085, 95% CI 90.922-410.041), poor interpersonal relationships (OR 34.195, 95% CI 25.824-45.280), poor stress management (OR 33.339, 95% CI 26.0259-42.710), poor self-realization (OR 11.504, 95% CI 9.371-14.122)), poor health responsibility (OR 9.828, 95% CI 6.072-15.909) and low levels of sports and exercise (OR 6.526, 95% CI 5.233-8.139).

**Table 4:** **Associations between breakfast eating frequency and health-promoting lifestyle**

**Dependent variables**	**Breakfast eating habits**					
	**Scarcely**			**Sometimes**		
	**B** ^**a**^	**OR (95% CI)**	**p-value**	**B** ^**a**^	**OR (95% CI)**	**p-value**
Health-promoting lifestyle						
Poor	4.558	95.415 (58.489-155.654)	0.000	2.473	11.855 (8.839-15.900)	0.000
General	3.095	22.077 (13.92-35.004)	0.000	1.914	6.781 (5.375-8.555)	0.000
Good	1.692	5.430 (3.410-8.645)	0.000	1.235	3.438 (2.720-4.345)	0.000
Excellent	Reference			Reference		
Self-realization						
Poor	2.443	11.504 (9.371-14.122)	0.000	1.098	2.999 (2.424-3.711)	0.000
General	1.624	5.071 (4.505-5.5.708)	0.000	1.067	2.905 (2.644-3.193)	0.000
Good	0.696	2.006 (1.787-2.251)	0.000	0.714	2.042 (1.873-2.225)	0.000
Excellent	Reference			Reference		
Health responsibility						
Poor	2.285	9.828 (6.072-15.909)	0.000	1.495	4.458 (3.296-6.030)	0.000
General	1.943	6.979 (4.316-11.285)	0.000	1.376	3.959 (2.933-5.344)	0.000
Good	1.393	4.025 (2.452-6.608)	0.000	1.270	3.562 (2.609-4.862)	0.000
Excellent	Reference			Reference		
Sports and exercise						
Poor	1.876	6.526 (5.233-8.139)	0.000	1.331	3.785 (3.189-4.493)	0.000
General	1.516	4.555 (3.673-5.649)	0.000	1.240	3.457 (2.933-4.075)	0.000
Good	0.636	1.890 (1.504-2.374)	0.000	0.902	2.464 (2.078-2.924)	0.000
Excellent	Reference			Reference		
Nutrition						
Poor	5.263	193.085 (90.922-410.041)	0.000	2.671	14.458 (10.797-19.361)	0.000
General	3.860	47.448 (22.475-100.170)	0.000	2.254	9.522 (7.254-12.497)	0.000
Good	2.038	7.677 (3.614-16.307)	0.000	1.385	3.994 (3.033-5.259)	0.000
Excellent	Reference			Reference		
Interpersonal relationship						
Poor	3.532	34.195 (25.824-45.280)	0.000	1.956	7.070 (5.297-9.436)	0.000
General	1.952	7.041 (6.022-8.233)	0.000	1.471	4.353 (3.864-4.903)	0.000
Good	0.874	2.397 (2.052-2.799)	0.000	1.018	2.768 (2.471-3.100)	0.000
Excellent	Reference			Reference		
Stress management						
Poor	3.507	33.339 (26.0259-42.710)	0.000	1.723	5.600 (4.363-7.187)	0.000
General	2.285	9.826 (8.353-11.559)	0.000	1.580	4.857 (4.320-5.460)	0.000
Good	1.069	2.914 (2.476-3.429)	0.000	1.127	3.086 (2.758-3.453)	0.000
Excellent	Reference			Reference		

Regressions conducted by iteratively regressing one health-promoting lifestyle on breakfast eating habits.

^a^Unstandardized regression coefficients.

The model is adjusted for demographic variables include age, gender, BMI, married status, education level, occupation, drinking, and smoking.

### Association between breakfast eating habits and health status

Odds ratios pertaining to breakfast eating frequency and health status are presented in Table 5. Positive frequency-responses were noted with respect to the likelihood of both SHS and disease in concurrence with breakfast eating frequency. There were lower levels of breakfast consumption regularity amongst individuals with SHS than those with disease. Those respondents reporting a scarce level of frequency were almost three times more likely to contract SHS (odds ratio 2.745, 95% confidence interval 2.468 to 3.053), while those citing infrequent breakfast consumption were approximately 1.7 times more likely to contract SHS (OR 1.731, 95% CI 1.879-21.595), relative to habitual breakfast eaters (p <0.001). Similarly, the group characterized by the lowest frequency was approximately 88% more likely to contract disease than habitual breakfast eaters (OR 1.878, 95% CI 1.673-2.108). There were significant effects on the likelihood of SHS with respect to three symptomatic dimensions (physiological, psychological and social); for example, compared with habitual breakfast eaters, individuals with the lowest breakfast eating frequency were approximately 63% more likely to exhibit physiological SHS (OR 1.625, 95% CI 1.468-1.800), while odds ratios of 1.588 (95% CI 1.412-1.786) and 1.83 (95% CI 1.612-2.078) were associated with physiological SHS and social SHS, respectively, within the same group. Results remained consistent throughout analyses, with all evidence indicating that breakfast consumption frequency (dependent variable) has significant associations with self-reported symptoms when the three aforementioned dimensions are employed as model outcomes (Additional file 1: Tables S1, S2 and S3 in the Data Supplement).

**Table 5:** **Odds ratios pertaining to health status and breakfast eating frequency via multinomial logistic regression modeling**

**Independent variables**	**Breakfast eating frequency**						
	**Scarcely**			**Sometimes**			**Always**
	**B** ^**a**^	**OR (95% CI)**	**p-value**	**B** ^**a**^	**OR (95% CI)**	**p-value**	
SHS	1.010	2.745 (2.468-3.053)	0.000	0.549	1.731 (1.595-1.879)	0.000	Reference
Physiological	0.486	1.625 (1.468-1.800)	0.000	0.238	1.268 (1.158-1.389)	0.000	Reference
Physiological	0.463	1.588 (1.412-1.786)	0.000	0.270	1.310 (1.182-1.451)	0.000	Reference
Social	0.605	1.830 (1.612-2.078)	0.000	0.352	1.422 (1.280-1.579)	0.000	Reference
Disease	0.630	1.878 (1.673-2.108)	0.000	0.294	1.341 (1.227-1.466)	0.000	Reference

^a^Unstandardized regression coefficients.

The model is adjusted for demographic variables include age, gender, BMI, married status, education level, occupation, drinking, and smoking.

## Discussion

To date, the current study represents the first comprehensive investigation of the associations between healthy lifestyle, SHS and breakfast eating habits within the Chinese population. Findings indicate that irregular breakfast eating is associated with myriad unhealthy lifestyle behaviors. A significant association was found between the frequency of breakfast consumption and the prevalence of both SHS and disease; a frequency-response effect was noted, with increased breakfast consumption being correlated with decreased prevalence of both conditions. Habits pertaining to breakfast consumption were significantly associated with all three health status dimensions (physiological, psychological and social).

### Breakfast eating habits and demographic characteristics

The lowest level of breakfast consumption was observed amongst <25 years of age, with breakfast skipping increasing in prevalence with the transition to adulthood. Overall, results indicate that adolescents are more likely to skip breakfast, with similar findings having been previously reported [17,33]. Females, married individuals and those with higher levels of education exhibited more frequent breakfast eating habits; similar findings have been reported within a nationally representative UK sample [3]. Moreover, analyses revealed that categorically unhealthy behaviors such as smoking or drinking were frequently accompanied by a decreasing frequency of breakfast consumption. Further studies and resulting strategies are required to communicate the importance of breakfast eating in order to effectively meet the health requirements of specific population demographics.

### Breakfast eating habits and health-promoted lifestyle

Findings indicate a significant relationship between breakfast consumption and participant lifestyle. Those who frequently consumed breakfast tended to be associated with healthier overall lifestyle patterns, including elevated attention towards personal nutrition, enhanced interpersonal relationships and stress management capabilities and frequent physical activity. For example, habitual breakfast eaters exhibited a higher proclivity towards physical activities than infrequent breakfast eaters [3], while those that often skipped breakfast were associated with a higher daily intake of fat, cholesterol and energy than habitual breakfast eaters [34]. Assessment of breakfast eating habits may represent a powerful tool for the prediction of a healthy lifestyle.

### Breakfast eating habits and SHS

The World Health Organization (WHO) has defined health as “a state of complete physical, mental and social well-being and not merely the absence of disease or infirmity” since 1948 [35]. This concept is put forward from the high pace of life of the body and mental reaction and people's emphasis on quality of life. But this definition is still a controversy for in the research of intension about health, there are problems as obscurities in definition [36]. The controversy, however, drove the need for a definition and a deeper understanding of the intermediate state between health and disease, that’s what is called SHS, which have been put forward to account for it by Wang [27,28,37]. Results in this study indicate a high prevalence of SHS among the sample population (46%), which is similar to previous reports [24,38]. High though the prevalence of SHS is, the mechanism underlying SHS has yet to be ascertained and there has been a lack of objective clinical diagnostics for SHS. SHS questionnaires have been developed and widely used as diagnostic instruments of SHS in China, such as Suboptimal Health Status Questionnaire-25 (SHSQ-25) [39,40] and Multidimensional Sub-health Questionnaire of Adolescents (MSQA) [41]. SHSQ-25 is mainly aimed at physiological and psychological SHS and MSQA mainly suitable for adolescents. SHMS V1.0, however, is a multidimensional, self-report symptom inventory including physiological, psychological and social dimensions [30], which corresponds to the greater understanding of WHO’s definition about health. Since the mechanism underlying SHS has yet to be ascertained, the objective measurements for SHS are in the condition of exploration. Li, G., *et al*. [42] have investigated the biomarkers of SHS being attributable to the oxidative stress perspective, and found that SHS was associated with the plasma levels of thiobarbituric acid-reactive substances (TBARS).

Data obtained during the current study exhibit a marked association between SHS prevalence, disease and breakfast consumption frequency, with a positive frequency response evident between the likelihood of both SHS and disease contraction with breakfast eating habits i.e. decreased consumption frequency concurrent with increased likelihood of an adverse health response. Overall, SHS was more evident than disease within the sample population. Moreover, the positive association found between breakfast consumption habits and the instance of disease confirms results of previous studies [34], thus calling for further investigation of the association between breakfast consumption behaviors and the instance of *SHS*. Furthermore, results revealed a significant association between breakfast eating habits and assessed health status with respect to three symptomatic dimensions (physiological, psychological and social), based upon 35 existing symptom definitions from SHMS V1.0. Previous studies have reported that breakfast consumption is related to psychological variables, including eating style (cognitive restraint, uninhibited eating and emotional eating) [3]; this may be explanatory with respect to the aforementioned associations with both psychological and social health.

Tanaka *et al*. [43] have found that skipping breakfast was associated with an elevated prevalence of fatigue, while Smith *et al*. [44] provide evidence that consumption of high fibre cereals could represent a simple method for fatigue reduction. Smith *et al*. [45] have also shown that habitual breakfast consumption is was associated with a lower prevalence with respect to the common cold. Lawton *et al*. [46] previously reported that consumption of high fibre breakfast cereals may improve self-assessed digestive functionality and general wellbeing.

Meal timing may be causative with respect to the metabolic effects leading to SHS or disease; previous studies have shown that skipping breakfast may reduce insulin sensitivity in response to pre-loading, thus disturbing lipid profiles [10,47]. Further, omission of breakfast has been associated with impacts on overall diet composition via disruption of daily energy intake and up-regulation of appetite later in the day, leading to deleterious metabolic and endocrine-related variation in response to food consumed later in the morning [1,47,48]. Accordingly, less frequent eaters exhibit an inherently higher likelihood of contracting chronic diseases including diabetes, obesity and cardiovascular disease [34,49] in addition to SHS, thus further emphasizing the need for regular breakfast consumption.

### Healthy lifestyles, SHS and breakfast eating habits

A potential reason for lower levels of breakfast consumption regularity amongst individuals with SHS than those with disease may be an inherently higher appreciation of the importance of lifestyle among the latter group. This hypothesis is supported by previous findings which have shown that breakfast consumption is correlated with individual consciousness [3].

An improved understanding of the role of breakfast consumption behaviors may have broad applications in public health and health promotion; the study demonstrates that breakfast eating behaviors may be an accurate behavioral marker for healthy lifestyle promotion, particularly in terms of SHS or disease. Previous studies have shown that underlying health-related risk factors include increased psychological pressure, poor dietary habits, sleep deprivation, social competition, sedentary activities, smoking and alcohol abuse [25,50-52], thus supporting findings from the current study. Our results suggest that habitual breakfast consumption leads to improved health and wellbeing via individual consciousness of healthy lifestyle decisions. Further studies should further examine breakfast food types and quality, and address these potential mechanisms of SHS and disease at the individual or population level via larger and longer trials.

### Study strengths and limitations

The current study represents the first comprehensive investigation of associations between breakfast eating habits and SHS within the Chinese population. Potential confounders have been accounted for via consideration of baseline sample characteristics. Furthermore, this study is the first to demonstrate that breakfast eating behaviors are significantly associated with physiological, psychological and social health.

As with any respondent-completed questionnaire survey, responses may comprise a level of inherent inaccuracy or bias. The survey sample was comprised of a disproportionate number of students, thus representing a potential lack of population representivity. Moreover, although adjustments were made for several factors potentially associated with health and lifestyle, data pertaining to all variables related with breakfast eating were not collated (e.g. breakfast food type and quality), and were thus not included for analyses; future research should address these research limitations.

## Conclusions

The presented cross-sectional survey is the first to assess breakfast eating habits in relation to healthy lifestyles and suboptimal health status within the Chinese population; highlighted associations are significant. Breakfast eating behaviors were found to be associated with suboptimal health status within three distinct categories, namely physiological, psychological and social health. Study findings provide evidence which supports regular breakfast consumption; appropriate dietary habits may contribute to reducing the prevalence of SHS and associated disease at both the individual and population levels in China, thus leading to healthier lifestyles and the associated social and economic benefits.

## Additional file

Additional file 1:
**Associations between the self-report symptoms of SHMS V1.0 and breakfast eating.** **Table S1.** Odds of the self-report physiological symptoms of SHMS V1.0 for the risk profile groups of breakfast eating. **Table S2.** Odds of the self-report physiological symptoms of SHMS V1.0 for the risk profile groups of breakfast eating. **Table S3.** Odds of the self-report social symptoms of SHMS V1.0 for the risk profile groups of breakfast eating.
