# Supplementary material for: Associations between breakfast eating habits and health-promoting lifestyle, suboptimal health status in Southern China: a population based, cross sectional study
Source: J Transl Med. 2014 Dec 11;12:348. doi: 10.1186/s12967-014-0348-1 (PMC4269950; doi:10.1186/s12967-014-0348-1)
Supplement: Additional file 1: — Associations between the self-report symptoms of SHMS V1.0 and breakfast eating. Table S1. Odds of the self-report physiological symptoms of SHMS V1.0 for the risk profile groups of breakfast eating. Table S2. Odds of the self-report physiological symptoms of SHMS V1.0 for the risk profile groups of breakfast eating. Table S3. Odds of the self-report social symptoms of SHMS V1.0 for the risk profile groups of breakfast eating. [file 12967_2014_348_MOESM1_ESM.docx]

**Associations between the self-report symptoms of SHMS V1.0 and breakfast eating**

**Table S1. Odds of the self-report physiological symptoms of SHMS V1.0 for the risk profile groups of breakfast eating**

| physiological symptoms | Breakfast eating habits | | | | | | |
| --- | --- | --- | --- | --- | --- | --- | --- |
|  | Scarcely | | |  | Sometimes | | |
|  | B ^a^ | OR(95%CI) | p-value |  | B ^a^ | OR(95%CI) | p-value |
| How about your appetite? | | | | | | | |
| very poor | 1.291 | 3.637(2.677-4.941) | 0.000 |  | 0.342 | 1.407(1.006-1.969) | 0.046 |
| poor | 1.351 | 3.862(3.215-4.638) | 0.000 |  | 0.970 | 2.637(2.233-3.113) | 0.000 |
| general | 0.825 | 2.282(2.036-2.556) | 0.000 |  | 0.690 | 1.993(1.813-2.191) | 0.000 |
| good | 0.199 | 1.220(1.086-1.371) | 0.001 |  | 0.386 | 1.471(1.340-1.616) | 0.000 |
| very good | Reference | | |  | Reference | | |
| How about your sleep? | | | | | | | |
| very poor | 1.202 | 3.328(2.634-4.204) | 0.000 |  | 0.514 | 1.671(1.336-2.090) | 0.000 |
| poor | 0.930 | 2.534(2.178-2.949) | 0.000 |  | 0.576 | 1.778(1.566-2.020) | 0.000 |
| general | 0.737 | 2.091(1.826-2.393) | 0.000 |  | 0.596 | 1.814(1.626-2.023) | 0.000 |
| good | 0.191 | 1.210(1.051-1.394) | 0.008 |  | 0.319 | 1.376(1.230-1.539) | 0.000 |
| very good | Reference | | |  | Reference | | |
| Are you satisfied with your hair growth? | | | | | | | |
| never | 0.767 | 2.153(1.843-2.515) | 0.000 |  | 0.405 | 1.499(1.308-1.718) | 0.000 |
| little | 0.597 | 1.816(1.582-2.084) | 0.000 |  | 0.540 | 1.715(1.530-1.923) | 0.000 |
| general | 0.695 | 2.004(1.778-2.259) | 0.000 |  | 0.528 | 1.696(1.533-1.875) | 0.000 |
| good | 0.328 | 1.388(1.231-1.564) | 0.000 |  | 0.365 | 1.440(1.304-1.589) | 0.000 |
| very good | Reference | | |  | Reference | | |
| Do you suffer from palpitations, chest tightness, or shortness of breath? | | | | | | | |
| never | Reference | | |  | Reference | | |
| occasionally | 0.143 | 1.154(1.011-1.317) | 0.034 |  | 0.390 | 1.477(1.317-1.656) | 0.046 |
| sometimes | 0.442 | 1.555(1.367-1.770) | 0.000 |  | 0.537 | 1.711(1.529-1.915) | 0.000 |
| constantly | 0.554 | 1.740(1.476-2.053) | 0.000 |  | 0.442 | 1.556(1.344-1.803) | 0.000 |
| always | 0.703 | 2.019(1.424-2.862) | 0.001 |  | 0.408 | 1.503(1.076-2.101) | 0.017 |
| Do you suffer from gastrointestinal discomfort? | | | | | | | |
| never | Reference | | |  | Reference | | |
| occasionally | 0.167 | 1.182(1.047-1.335) | 0.007 |  | 0.259 | 1.295(1.166-1.439) | 0.046 |
| sometimes | 0.438 | 1.550(1.373-1.749) | 0.000 |  | 0.426 | 1.531(1.378-1.700) | 0.000 |
| constantly | 0.488 | 1.628(1.396-1.899) | 0.000 |  | 0.327 | 1.386(1.213-1.584) | 0.000 |
| always | 0.494 | 1.638(1.262-2.127) | 0.001 |  | 0.001 | 1.001(0.782-1.280) | 0.995 |
| Do you suffer from abnormal urine? | | | | | | | |
| never | Reference | | |  | Reference | | |
| occasionally | 0.183 | 1.200(1.089-1.323) | 0.000 |  | 0.231 | 1.260(1.161-1.367) | 0.000 |
| sometimes | 0.332 | 1.394(1.260-1.542) | 0.000 |  | 0.323 | 1.382(1.268-1.506) | 0.000 |
| constantly | 0.330 | 1.390(1.171-1.651) | 0.000 |  | 0.390 | 1.477(1.278-1.706) | 0.000 |
| always | 0.684 | 1.981(1.413-2.777) | 0.001 |  | 0.309 | 1.362(0.990-1.873) | 0.058 |
| Do you suffer from head discomfort? | | | | | | | |
| never | Reference | | |  | Reference | | |
| occasionally | 0.293 | 1.340(1.205-1.491) | 0.000 |  | 0.290 | 1.336(1.221-1.463) | 0.000 |
| sometimes | 0.568 | 1.764(1.582-1.967) | 0.000 |  | 0.469 | 1.598(1.457-1.753) | 0.000 |
| constantly | 0.690 | 1.994(1.706-2.331) | 0.000 |  | 0.485 | 1.624(1.420-1.856) | 0.000 |
| always | 0.978 | 2.660(1.862-3.801) | 0.000 |  | 0.249 | 1.283(0.894-1.841) | 0.176 |
| Are you suffering from eye discomfort? | | | | | | | |
| never | Reference | | |  | Reference | | |
| occasionally | 0.214 | 1.239(1.104-1.390) | 0.000 |  | 0.241 | 1.273(1.153-1.405) | 0.000 |
| sometimes | 0.393 | 1.481(1.326-1.655) | 0.000 |  | 0.314 | 1.368(1.244-1.505) | 0.000 |
| constantly | 0.345 | 1.412(1.235-1.616) | 0.000 |  | 0.285 | 1.330(1.186-1.492) | 0.000 |
| always | 0.390 | 1.477(1.132-1.927) | 0.004 |  | 0.131 | 1.140(0.897-1.449) | 0.284 |
| Do you suffer hearing system abnormalities? | | | | | | | |
| never | Reference | | |  | Reference | | |
| occasionally | 0.356 | 1.427(1.314-1.550) | 0.000 |  | 0.355 | 1.426(1.330-1.527) | 0.000 |
| sometimes | 0.563 | 1.755(1.594-1.934) | 0.000 |  | 0.379 | 1.461(1.344-1.588) | 0.000 |
| constantly | 0.576 | 1.779(1.452-2.180) | 0.000 |  | 0.375 | 1.456(1.217-1.741) | 0.000 |
| always | 0.268 | 1.307(0.928-1.840) | 0.125 |  | -0.099 | 0.905(0.657-1.248) | 0.543 |
| Do you have difficulty with your knees or with bending over? | | | | | | | |
| never | Reference | | |  | Reference | | |
| little | 0.414 | 1.513(1.387-1.650) | 0.000 |  | 0.272 | 1.313(1.219-1.414) | 0.000 |
| some | 0.845 | 2.328(2.055-2.637) | 0.000 |  | 0.376 | 1.457(1.299-1.633) | 0.000 |
| hard | 0.911 | 2.486(1.831-3.374) | 0.000 |  | 0.277 | 1.319(0.978-1.777) | 0.069 |
| very hard | 0.782 | 2.186(1.114-4.291) | 0.023 |  | 0.601 | 1.823(1.012-3.285) | 0.046 |
| Do you have any difficulty in climbing 3–5 floors? | | | | | | | |
| never | Reference | | |  | Reference | | |
| little | 0.463 | 1.589(1.457-1.734) | 0.000 |  | 0.236 | 1.266(1.175-1.364) | 0.000 |
| some | 0.831 | 2.295(2.044-2.576) | 0.000 |  | 0.364 | 1.438(1.298-1.594) | 0.000 |
| hard | 1.096 | 2.992(2.128-4.207) | 0.000 |  | 0.648 | 1.913(1.406-2.601) | 0.000 |
| very hard | 1.143 | 3.136(1.688-5.828) | 0.000 |  | 0.155 | 1.168(0.598-2.281) | 0.649 |
| Do you have any difficulty in walking 1500 m? | | | | | | | |
| never | Reference | | |  | Reference | | |
| little | 0.505 | 1.658(1.515-1.814) | 0.000 |  | 0.319 | 1.376(1.274-1.486) | 0.000 |
| some | 0.955 | 2.598(2.302-2.932) | 0.000 |  | 0.502 | 1.653(1.481-1.844) | 0.000 |
| hard | 0.956 | 2.601(1.983-3.412) | 0.000 |  | 0.383 | 1.466(1.132-1.898) | 0.004 |
| very hard | 0.837 | 2.310(1.424-3.746) | 0.001 |  | 0.385 | 1.469(0.933-2.312) | 0.097 |
| Could the fatigue be alleviated by rest? | | | | | | | |
| never | 0.778 | 2.178(1.684-2.818) | 0.000 |  | 0.187 | 1.206(0.934-1.558) | 0.152 |
| occasionally | 1.591 | 4.911(3.976-6.065) | 0.000 |  | 0.833 | 2.301(1.859-2.848) | 0.000 |
| sometimes | 1.286 | 3.618(3.212-4.075) | 0.000 |  | 0.685 | 1.983(1.774-2.217) | 0.000 |
| constantly | 0.452 | 1.571(1.451-1.702) | 0.000 |  | 0.421 | 1.523(1.428-1.626) | 0.000 |
| always | Reference | | |  | Reference | | |
| Do you have enough energy to cope with everyday life, work and learn? | | | | | | | |
| never | 1.178 | 3.248(2.508-4.207) | 0.000 |  | 0.475 | 1.608(1.237-2.090) | 0.000 |
| occasionally | 1.400 | 4.053(3.446-4.768) | 0.000 |  | 0.829 | 2.292(1.963-2.676) | 0.000 |
| sometimes | 1.184 | 3.268(2.921-3.657) | 0.000 |  | 0.683 | 1.980(1.789-2.191) | 0.000 |
| constantly | 0.405 | 1.499(1.373-1.637) | 0.000 |  | 0.427 | 1.533(1.427-1.646) | 0.000 |
| always | Reference | | |  | Reference | | |

^a^ Unstandardized regression coefficients.

The model is adjusted for demographic variables, including age, gender, BMI, married status, education level, occupation, drinking, and smoking.

**Table S2. Odds of the self-report physiological symptoms of SHMS V1.0 for the risk profile groups of breakfast eating**

| Psychological symptoms | Breakfast eating habits | | | | | | |
| --- | --- | --- | --- | --- | --- | --- | --- |
|  | Scarcely | | |  | Sometimes | | |
|  | B ^a^ | OR(95%CI) | p-value |  | B ^a^ | OR(95%CI) | p-value |
| Do you have confidence? | | | | | | | |
| never | 1.492 | 4.446(3.203-6.170) | 0.000 |  | 0.437 | 1.548(1.078-2.223) | 0.018 |
| occasionally | 1.013 | 2.754(2.403-3.156) | 0.000 |  | 0.556 | 1.744(1.542-1.973) | 0.000 |
| some | 0.758 | 2.133(1.934-2.353) | 0.000 |  | 0.486 | 1.626(1.495-1.770) | 0.000 |
| much | 0.193 | 1.213(1.104-1.333) | 0.000 |  | 0.277 | 1.319(1.221-1.424) | 0.000 |
| quite | Reference | | |  | Reference | | |
| Are you satisfied with your living conditions? | | | | | | | |
| never | 1.271 | 3.563(2.892-4.389) | 0.000 |  | 0.776 | 2.173(1.790-2.639) | 0.000 |
| occasionally | 1.149 | 3.155(2.655-3.748) | 0.000 |  | 0.900 | 2.459(2.124-2.847) | 0.000 |
| general | 0.994 | 2.703(2.345-3.114) | 0.000 |  | 0.825 | 2.281(2.034-2.558) | 0.000 |
| good | 0.355 | 1.427(1.237-1.646) | 0.000 |  | 0.504 | 1.656(1.480-1.852) | 0.000 |
| very good | Reference | | |  | Reference | | |
| Are you optimistic about the future? | | | | | | | |
| never | 1.141 | 3.131(2.409-4.068) | 0.000 |  | 0.512 | 1.668(1.283-2.169) | 0.000 |
| occasionally | 1.121 | 3.067(2.580-3.646) | 0.000 |  | 0.705 | 2.024(1.726-2.373) | 0.000 |
| some | 0.936 | 2.550(2.289-2.839) | 0.000 |  | 0.632 | 1.881(1.717-2.061) | 0.000 |
| much | 0.326 | 1.385(1.250-1.535) | 0.000 |  | 0.404 | 1.4971.379-1.626) | 0.000 |
| quite | Reference | | |  | Reference | | |
| Are you feeling happy? | | | | | | | |
| never | 1.319 | 3.740(2.936-4.764) | 0.000 |  | 0.493 | 1.637(1.269-2.112) | 0.000 |
| occasionally | 1.134 | 3.107(2.676-3.606) | 0.000 |  | 0.654 | 1.923(1.673-2.210) | 0.000 |
| sometimes | 0.903 | 2.466(2.231-2.726) | 0.000 |  | 0.691 | 1.996(1.836-2.171) | 0.000 |
| constantly | 0.279 | 1.322(1.191-1.468) | 0.000 |  | 0.427 | 1.532(1.410-1.664) | 0.000 |
| always | Reference | | |  | Reference | | |
| Do you feel nervous? | | | | | | | |
| never | Reference | | |  | Reference | | |
| occasionally | 0.086 | 1.090(0.941-1.263) | 0.250 |  | 0.338 | 1.403(1.228-1.602) | 0.000 |
| sometimes | 0.334 | 1.396(1.213-1.608) | 0.000 |  | 0.498 | 1.645(1.447-1.871) | 0.000 |
| constantly | 0.540 | 1.717(1.452-2.029) | 0.000 |  | 0.410 | 1.506(1.292-1.755) | 0.000 |
| always | 0.712 | 2.038(1.522-2.729) | 0.000 |  | 0.260 | 1.297(0.970-1.734) | 0.080 |
| Do you experience bad moods or depression? | | | | | | | |
| never | Reference | | |  | Reference | | |
| occasionally | 0.168 | 1.183(0.972-1.441) | 0.094 |  | 0.392 | 1.479(1.244-1.759) | 0.000 |
| sometimes | 0.622 | 1.863(1.542-2.251) | 0.000 |  | 0.683 | 1.980(1.674-2.341) | 0.000 |
| constantly | 0.939 | 2.558(2.077-3.149) | 0.000 |  | 0.781 | 2.183(1.813-2.629) | 0.000 |
| always | 1.354 | 3.873(2.782-5.394) | 0.000 |  | 0.664 | 1.943(1.388-2.720) | 0.000 |
| Do you feel insecure? | | | | | | | |
| never | Reference | | |  | Reference | | |
| occasionally | 0.226 | 1.253(1.140-1.377) | 0.000 |  | 0.325 | 1.384(1.278-1.500) | 0.000 |
| sometimes | 0.641 | 1.899(1.714-2.103) | 0.000 |  | 0.558 | 1.747(1.601-1.907) | 0.000 |
| constantly | 0.942 | 2.566(2.149-3.063) | 0.000 |  | 0.415 | 1.514(1.281-1.789) | 0.000 |
| always | 1.084 | 2.958(2.108-4.151) | 0.000 |  | 0.704 | 2.022(1.467-2.787) | 0.000 |
| Do you have no reason to feel afraid? | | | | | | | |
| never | Reference | | |  | Reference | | |
| occasionally | 0.376 | 1.456(1.338-1.583) | 0.000 |  | 0.372 | 1.450(1.353-1.555) | 0.000 |
| sometimes | 0.934 | 2.544(2.303-2.811) | 0.000 |  | 0.555 | 1.742(1.597-1.901) | 0.000 |
| constantly | 0.986 | 2.681(2.094-3.433) | 0.000 |  | 0.623 | 1.865(1.497-2.324) | 0.000 |
| always | 1.603 | 4.968(3.055-8.080) | 0.000 |  | 0.612 | 1.845(1.094-3.110) | 0.022 |
| Do you feel lonely? | | | | | | | |
| never | Reference | | |  | Reference | | |
| occasionally | 0.312 | 1.367(1.237-1.510) | 0.000 |  | 0.371 | 1.449(1.336-1.572) | 0.000 |
| some | 0.721 | 2.057(1.872-2.260) | 0.000 |  | 0.565 | 1.760(1.624-1.907) | 0.000 |
| much | 0.910 | 2.484(2.132-2.894) | 0.000 |  | 0.632 | 1.882(1.641-2.158) | 0.000 |
| quite | 1.029 | 2.798(2.135-3.667) | 0.000 |  | 0.422 | 1.526(1.157-2.011) | 0.003 |
| Are you sensitive or suspicious? | | | | | | | |
| never | Reference | | |  | Reference | | |
| occasionally | 0.249 | 1.283(1.161-1.417) | 0.000 |  | 0.331 | 1.393(1.280-1.515) | 0.000 |
| sometimes | 0.484 | 1.623(1.466-1.797) | 0.000 |  | 0.421 | 1.524(1.397-1.662) | 0.000 |
| constantly | 0.595 | 1.813(1.553-2.117) | 0.000 |  | 0.409 | 1.505(1.314-1.724) | 0.000 |
| always | 0.770 | 2.159(1.644-2.835) | 0.000 |  | -0.168 | 0.846(0.626-1.143) | 0.276 |
| How is your memory? | | | | | | | |
| very poor | 0.962 | 2.617(2.018-3.394) | 0.000 |  | 0.589 | 1.802(1.417-2.291) | 0.000 |
| poor | 0.793 | 2.210(1.811-2.697) | 0.000 |  | 0.639 | 1.894(1.588-2.258) | 0.000 |
| general | 0.483 | 1.620(1.348-1.948) | 0.000 |  | 0.542 | 1.719(1.462-2.020) | 0.000 |
| good | 0.121 | 1.129(0.934-1.365) | 0.211 |  | 0.336 | 1.400(1.187-1.652) | 0.000 |
| very good | Reference | | |  | Reference | | |
| What about your ability to think and solve problems? | | | | | | | |
| very poor | 1.642 | 5.164(3.555-7.501) | 0.000 |  | 0.995 | 2.704(1.881-3.889) | 0.000 |
| poor | 1.195 | 3.302(2.649-4.116) | 0.000 |  | 0.851 | 2.342(1.931-2.841) | 0.000 |
| general | 0.823 | 2.278(1.901-2.729) | 0.000 |  | 0.748 | 2.113(1.814-2.462) | 0.000 |
| good | 0.358 | 1.430(1.192-1.716) | 0.000 |  | 0.472 | 1.603(1.375-1.868) | 0.000 |
| very good | Reference | | |  | Reference | | |

^a^ Unstandardized regression coefficients.

The model is adjusted for demographic variables, including age, gender, BMI, married status, education level, occupation, drinking, and smoking.

**Table S3. Odds of the self-report social symptoms of SHMS V1.0 for the risk profile groups of breakfast eating**

| social symptoms | Breakfast eating habits | | | | | | |
| --- | --- | --- | --- | --- | --- | --- | --- |
|  | Scarcely | | |  | Sometimes | | |
|  | B ^a^ | OR(95%CI) | p-value |  | B ^a^ | OR(95%CI) | p-value |
| Can you appropriately deal with unhappy events in your life, work and school? | | | | | | | |
| never | 2.034 | 7.643(5.012-11.656) | 0.000 |  | 1.110 | 3.033(1.940-4.741) | 0.000 |
| occasionally | 1.586 | 4.886(3.975-6.006) | 0.000 |  | 0.781 | 2.184(1.774-2.690) | 0.000 |
| sometimes | 1.187 | 3.276(2.904-3.696) | 0.000 |  | 0.755 | 2.129(1.914-2.367) | 0.000 |
| constantly | 0.327 | 1.387(1.252-1.537) | 0.000 |  | 0.430 | 1.537(1.412-1.673) | 0.000 |
| always | Reference | | |  | Reference | | |
| Are you satisfied with your social relationships? | | | | | | | |
| never | 1.233 | 3.433(2.589-4.552) | 0.000 |  | 0.778 | 2.177(1.634-2.900) | 0.000 |
| rarely | 0.989 | 2.690(2.236-3.235) | 0.000 |  | 0.941 | 2.561(2.163-3.033) | 0.000 |
| general | 0.813 | 2.254(1.945-2.612) | 0.000 |  | 0.901 | 2.462(2.154-2.814) | 0.000 |
| good | 0.280 | 1.323(1.142-1.533) | 0.000 |  | 0.710 | 2.034(1.783-2.319) | 0.000 |
| very good | Reference | | |  | Reference | | |
| Are you satisfied with your performance in your life, work and school? | | | | | | | |
| never | 1.415 | 4.119(3.068-5.529) | 0.000 |  | 1.009 | 2.742(2.066-3.639) | 0.000 |
| rarely | 1.286 | 3.617(2.956-4.426) | 0.000 |  | 1.014 | 2.757(2.302-3.301) | 0.000 |
| general | 0.952 | 2.590(2.182-3.075) | 0.000 |  | 0.949 | 2.582(2.223-2.998) | 0.000 |
| good | 0.256 | 1.291(1.086-1.536) | 0.004 |  | 0.611 | 1.842(1.587-2.138) | 0.000 |
| very good | Reference | | |  | Reference | | |
| Can you quickly adapt to new living, working and learning environments? | | | | | | | |
| never | 1.915 | 6.790(4.366-10.559) | 0.000 |  | 0.889 | 2.433(1.516-3.906) | 0.000 |
| occasionally | 1.485 | 4.413 (3.597-5.414) | 0.000 |  | 0.821 | 2.272(1.868-2.763) | 0.000 |
| sometimes | 1.406 | 4.081 (3.625-4.594) | 0.000 |  | 0.784 | 2.189(1.969-2.435) | 0.000 |
| constantly | 1.406 | 4.081 (3.625-4.594) | 0.000 |  | 0.784 | 2.189(1.969-2.435) | 0.000 |
| always | Reference | | |  | Reference | | |
| Do you always keep in touch with friends and family? | | | | | | | |
| never | 2.109 | 8.242(4.978-13.646) | 0.000 |  | 0.756 | 2.130(1.197-3.788) | 0.000 |
| occasionally | 1.065 | 2.901(2.553-3.295) | 0.000 |  | 0.583 | 1.792(1.601-2.005) | 0.000 |
| sometimes | 0.863 | 2.371(2.135-2.633) | 0.000 |  | 0.632 | 1.882(1.727-2.050) | 0.000 |
| constantly | 0.427 | 1.533(1.376-1.707) | 0.000 |  | 0.458 | 1.581(1.453-1.720) | 0.000 |
| always | Reference | | |  | Reference | | |
| Do you have friends to share your happiness and sadness? | | | | | | | |
| never | 1.157 | 3.181(2.440-4.147) | 0.000 |  | 0.747 | 2.111(1.652-2.698) | 0.000 |
| few | 0.890 | 2.435(2.106-2.816) | 0.000 |  | 0.626 | 1.869(1.659-2.107) | 0.000 |
| some | 0.817 | 2.263(1.973-2.595) | 0.000 |  | 0.657 | 1.930(1.728-2.155) | 0.000 |
| many | 0.399 | 1.491(1.296-1.714) | 0.000 |  | 0.458 | 1.580(1.415-1.764) | 0.000 |
| Very many, more | Reference | | |  | Reference | | |
| Do you have many colleagues, classmates, neighbours, relatives or friends close to you? | | | | | | | |
| never | 1.947 | 7.005(4.481-10.949) | 0.000 |  | 0.847 | 2.333(1.437-3.788) | 0.001 |
| few | 0.957 | 2.603(2.273-2.981) | 0.000 |  | 0.553 | 1.739(1.551-1.950) | 0.000 |
| some | 0.817 | 2.265(2.016-2.544) | 0.000 |  | 0.592 | 1.807(1.646-1.983) | 0.000 |
| many | 0.458 | 1.581(1.402-1.784) | 0.000 |  | 0.421 | 1.524(1.386-1.676) | 0.000 |
| Very many, more | Reference | | |  | Reference | | |
| When you need help, would your family, colleagues or friends provide physical or emotional support or help? | | | | | | | |
| never | 1.878 | 6.538(4.425-9.660) | 0.000 |  | 0.550 | 1.734(1.107-2.717) | 0.016 |
| occasionally | 1.573 | 4.820(4.040-5.750) | 0.000 |  | 0.885 | 2.422(2.050-2.861) | 0.000 |
| sometimes | 1.377 | 3.963(3.518-4.464) | 0.000 |  | 0.837 | 2.310(2.089-2.555) | 0.000 |
| constantly | 0.596 | 1.815(1.639-2.010) | 0.000 |  | 0.566 | 1.761(1.628-1.906) | 0.000 |
| always | Reference | | |  | Reference | | |
| When you are in trouble, would you seek support and help from others? | | | | | | | |
| never | 1.439 | 4.216(3.064-5.803) | 0.000 |  | 0.539 | 1.714(1.243-2.363) | 0.001 |
| occasionally | 0.982 | 2.670(2.148-3.319) | 0.000 |  | 0.767 | 2.153(1.799-2.578) | 0.000 |
| sometimes | 0.748 | 2.113(1.716-2.603) | 0.000 |  | 0.768 | 2.156(1.820-2.553) | 0.000 |
| constantly | 0.386 | 1.471(1.189-1.820) | 0.000 |  | 0.520 | 1.681(1.416-1.996) | 0.000 |
| always | Reference | | |  | Reference | | |

^a^ Unstandardized regression coefficients.

The model is adjusted for demographic variables, including age, gender, BMI, married status, education level, occupation, drinking, and smoking.
